# Dealing with resistance and negative attitudes as barriers to the implementation of a longitudinal communication curriculum – a field report

**DOI:** 10.3205/zma001447

**Published:** 2021-02-15

**Authors:** Anke Hollinderbäumer, B. Hinding, H. Buggenhagen, J. Jünger

**Affiliations:** 1 University Medical Center of the Johannes Gutenberg University Mainz, Rudolf Frey Lernklinik central learning platform, Mainz, Germany; 2 Institut für medizinische und pharmazeutische Prüfungsfragen (IMPP), Mainz, Germany

**Keywords:** resistance, negativ attitude, communication curriculum, implemention barriers

## Abstract

**Background: **The aim of the project “Communicative Competences of Physicians” (BMG) was the pilot implementation of a longitudinal model communication curriculum. For the Mainz project location, the task was to integrate courses offered in the clinical subjects into a communication curriculum and to expand it longitudinally. In this process, which was both content-related and social, resistance and negative attitudes regarding the continued development of the communication curriculum became apparent at an early stage. How these manifested and which measures were taken to overcome them is laid out in this reflective field report.

**Method/approach:** At the beginning of the project, a SWOT analysis was used to identify the strengths and weaknesses that the faculty presented for the continued development of the communication curriculum. This assessment by the project staff included, among other things, an evaluation of the motivation levels of the faculty’s lecturers and senior teaching staff. The subsequent specific, strategic and content-related planning of the further implementation steps in accordance with change-management concepts made it possible to take this aspect into account. For a more reliable assessment of the situation, the project was first presented to the faculty’s teaching committee. In this situation it was possible to identify individuals with favourable and unfavourable attitudes. With the insight that was gained, the following course of action was decided upon:

- Contact advocates to gain their support.
- Contact the individuals with negative attitudes with the aim of building a relationship and arranging a personal meeting.
- Identify resistances and negative attitudes in one-on-one meetings and employ targeted countermeasures.
- Develop an action plan with a particular focus on gaining the cooperation of those who are essential to the success of integrating communication-related content into the major clinical disciplines.

**Results/experiences: **In one-on-one meetings, it was possible to first clarify which learning objectives of communication training are already covered in the respective subject and which expertise is therefore available. Furthermore, it was possible to clarify which areas that were still absent in the overall communication curriculum should be taught in this clinical subject in particular. It became possible to involve the lecturers in the development in the spirit of participatory design.

In accordance with the action plan, offers to support the development and organisation of as-yet absent portions of the curriculum were presented to the affected departments.

**Discussion/conclusion: **Resistance and negative attitudes often do not represent a rejection of communicative competences – they rather express that teachers fear they do not have the expertise and resources to teach them. With the selected approach of outreach, personal conversation, and action plan, it was possible to provide the individuals in question with goal-oriented support.

## Zusammenfassung

**Hintergrund: **Das Projekt „Kommunikative Kompetenzen von Ärztinnen und Ärzten“ (BMG) hatte die Pilotimplementierung des longitudinalen Mustercurriculums Kommunikation zum Ziel. Für den Projektstandort Mainz stellte sich hierbei die Aufgabe, Lehrveranstaltungen aus den klinischen Fächern in ein Kommunikationscurriculum zu integrieren und dieses longitudinal auszuweiten. Bei diesem sowohl inhaltlichen als auch sozialen Prozess zeigten sich bereits früh Widerstände und negative Haltungen bezüglich der Weiterentwicklung des Kommunikationscurriculums. Die Erscheinungsformen und die zu ihrer Überwindung ergriffenen Maßnahmen sind Gegenstand des vorliegenden reflektierenden Erfahrungsberichts.

**Methode/Vorgehen: **Zu Beginn des Projekts wurden anhand einer SWOT-Analyse die Stärken und Schwächen identifiziert, welche die Fakultät für die Weiterentwicklung des Kommunikationscurriculums bietet. Diese Einschätzung der Projektmitarbeiter enthielt u.a. eine Beurteilung der Motivationslage der Dozierenden und Lehrverantwortlichen an der Fakultät. Die anschließende spezifische, strategische und inhaltliche Planung der weiteren Implementierungsschritte entlang von Change-Management-Konzepten gab die Möglichkeit, diesen Aspekt zu berücksichtigten. Für eine zuverlässigere Einschätzung der Situation wurde das Vorhaben zunächst im Fakultätsgremium Lehre vorgestellt. In dieser Situation war es möglich, Personen mit befürwortender und Personen mit ablehnender Haltung zu identifizieren. Mit der gewonnenen Erkenntnis wurde das folgende Vorgehen entschieden:

- Kontakt zu Befürworterinnen und Befürwortern aufnehmen, um ihre Unterstützung zu gewinnen.
- Kontakt zu den Personen mit ablehnender Haltung aufnehmen, mit dem Ziel des Beziehungsaufbaus und eines persönlichen Gesprächstermins.
- Widerstände und ablehnende Haltungen in Einzelgesprächen identifizieren und gezielte Gegenmaßnahmen anwenden.
- Aktionsplan erstellen, um insbesondere jene zur Zusammenarbeit zu gewinnen, die für das Gelingen der Integration kommunikationsbezogener Inhalte in die großen klinischen Fächer maßgeblich sind.

**Ergebnisse/Erfahrungen:** In Einzelgesprächen konnte zunächst geklärt werden, welche Lernziele der Kommunikationslehre im jeweiligen Fach bereits abgedeckt werden und welche Expertisen somit vorzufinden sind. Im Weiteren konnte erläutert werden, welche im Gesamtkommunikationscurriculum noch fehlenden Bereiche besonders in diesem klinischen Fach zu unterrichten wären. Die Dozierenden konnten im Sinne eines partizipatorischen Designs in die Entwicklungen eingebunden werden.

Dem Aktionsplan entsprechend konnten den betreffenden Fächern Unterstützungsangebote für die Entwicklung noch fehlender Lehreinheiten und deren Organisation vorgestellt werden.

**Diskussion/Fazit: **Widerstand und negative Haltungen stehen häufig nicht für die Ablehnung kommunikativer Kompetenzen, eher bringen sie zum Ausdruck, dass Lehrende fürchten, nicht über die Expertise und Ressourcen zu verfügen, diese zu vermitteln. Mit der entschiedenen Vorgehensweise: Kontaktaufnahme, persönliches Gespräch und Aktionsplan, konnten die betroffenen Personen zielführende Unterstützung erhalten.

## Background

Communicative competence as a key competence of professional medical interaction is insufficiently embedded in the training curricula of many faculties [1]. The development of the corresponding courses is a complex and multi-layered process. The primary aim of the project “Communicative Competences of Physicians” (BMG) was the pilot implementation of the longitudinal model communication curriculum [2]. The core task for Mainz, as one of four locations, was to integrate communication training into the clinical subjects and implement it longitudinally throughout the degree program.

It was known before the project began that there would be resistance and negative attitudes towards teaching communication at the location. Statements like: “That’s unnecessary” or “Students can do all of that already” reflected this. Ignoring resistance or countering it with social pressure results in obstruction and counter-pressure. Resistance overcome, on the other hand, can provide new resources [3]. For this reason, the following steps were taken.

## Method/procedure

At the outset of the project, the SWOT analysis [4], [5] was used to assess site-specific strengths and weaknesses for the continued development of the communication curriculum. The prevalence of a more positive or negative attitude [6], [7], [8] according to the concept of institutional readiness was observed. In particular, the strategic implementation stages according to the change management concepts of Lewin [9] and Kotter [10], [11] made it possible to consider this aspect. Lewin’s concept of “thawing” [9] emphasises that the affected individuals and the setting must be prepared for upcoming change processes. The aim is to achieve a broad-based conviction that change is needed, as well as emotional investment (enthusiasm). 

To allow for a realistic assessment of the situation, the project was presented to the faculty’s teaching committee. In this context, it was possible to reliably identify teachers and students with favourable and unfavourable attitudes, analyse the arguments that were presented, and evaluate them for further action. Based on the results, concrete courses of action were planned: 

### Contact supporters

An invitation to a group discussion was issued with the intention of finding support in this circle and allowing for reflection and coordination regarding the planned course of action.

#### Contact individuals with a negative attitude

For this group of people, a climate of trust had to be created as an important prerequisite for a successful change process, so that resistance could then be addressed openly in one-on-one conversations.

#### Identify resistance and negative attitudes and consciously manage the change process

The “four-room model” [12] describes how people typically experience change processes. Pressure to change is initially ignored, followed by a phase of confusion and only then acceptance and a willingness to actively participate. With this knowledge, reactions can be identified and people can be accompanied through change processes in a targeted manner. The recommendations of Kuster et al. [3] are also helpful. According to this, individuals are willing to change if, among other things:

- their past performance is recognised,
- they are well informed,
- the necessary support can be provided.

#### Create an action plan

Prior to the one-on-one meetings, supportive measures were listed that the neutral central learning platform Rudolf Frey Lernklinik can provide for communication training. These include:

- rooms for teaching communication in small groups, with associated observation booths,
- video technology for recording and analysing meetings,
- simulated patients,
- advice on the creation of teaching concepts.

## Results/experiences

In the SWOT analysis, positive and negative attitudes in terms of motivation levels were mentioned (see table 1 (Tab. 1)). Some particularly committed lecturers were named as an important resource, but scepticism and resistance were expected for the most part. The presentation to the faculty’s teaching committee made it possible to identify those in favour and those opposed.

Contact with supporters strengthened our position and yielded constructive advice on how to proceed, such as the opportunity for communication between lecturers on the evolution of teaching communication.

As a way of acknowledging past performance, individuals with negative attitudes were asked which learning objectives of the sample communication curriculum [2] were already mapped in their respective subjects. This was deliberately done in one-on-one conversations. In this way, information deficits could be identified and eliminated quickly. In this confidential environment, there was a greater willingness to address problems and deficits as well as the need for support. With the resulting action plan, it was possible to make offers that aligned with needs. In the spirit of participatory design [13], lecturers were successfully motivated to further develop communication instruction. Thus, ideas for the implementation of communication lessons arose spontaneously from the clinical subjects, e.g. to include informed-consent interviews in the practical course for surgery or prepare for parent interviews in paediatrics using peer interviews between students. In addition to this, the provided rooms with observation booths for small groups were well received, as were the simulated patients.

In the end, participation in communication instruction was secured for each clinical subject, and the model curriculum was expanded longitudinally. 

## Conclusion

The causes of resistance and negative attitudes are initially obscured. Often, these behaviours do not represent a rejection of the subject matter, but rather express that teachers believe they do not have enough expertise. With this knowledge, it was possible to provide the individuals in question with targeted information and needs-based support, thus persuading them to actively participate.

## Funding

This field report was developed as part of the project “Communicative competences of physicians – pilot implementation, accompanying evaluation and development of implementation strategies for a longitudinal model curriculum on communication in medicine” with funding from the German Federal Ministry of Health (BMG). Grant number: ZMV I1 2516FSB200. 

## Profiles

**Name of location: **University Medical Center of the Johannes Gutenberg University Mainz

**Field of study/profession: **Medicine

**Number of students per year or semester: **ca. 140-220 students per semester

**Has a longitudinal communication curriculum been implemented? **Yes

**In which semesters are communicative and social competences taught?** Semesters 1, 2, 4, 5, 6, 7, 8, 9, 10 

**What teaching formats are used?** Seminar, class, practical course, lecture, e-learning

**In which semesters are communicative and social competences tested (formative assessment or relevant for passing, and/or graded)? **Semesters 1, 2, 4, 5, 7, 8, 9, 10 

**Which examination formats are used? **Essay, key feature problem examination, poster production, oral-practical, OSCE, patient care report, open-book exam, multiple-choice exam

**Who (e.g. clinic, institution) is responsible for development and implementation?** Decentralised: each institution individually

## Current professional roles of the authors

Dr. rer. physiol. Anke Hollinderbäumer, MME

- graduate psychologist
- research assistant at the Rudolf Frey Lernklink central learning platform, responsible for the communication curriculum and OSCE examinations

Dr. phil. Barbara Hinding

- graduate psychologist
- research assistant at the Institute for Medical and Pharmaceutical Examination Questions (IMPP)

Dr. med. Holger Buggenhagen, MME

- head of the Rudolf Frey Klinik central learning platform
- senior anaesthesiologist

Prof. Dr. med. Jana Jünger, MME (Bern)

- Director of the Institute for Medical and Pharmaceutical Examination Questions (IMPP)
- development of the postgraduate program Master of Medical Education (MME) in Germany
- member of the MME course management and lecturer for the modules Examinations, Educational Research, and Evaluation
- management of various projects for the implementation of communication curricula in medical education as well as the development of new examination formats for the assessment of communicative competences

## Competing interests

The authors declare that they have no competing interests. 

## Figures and Tables

**Table 1:**
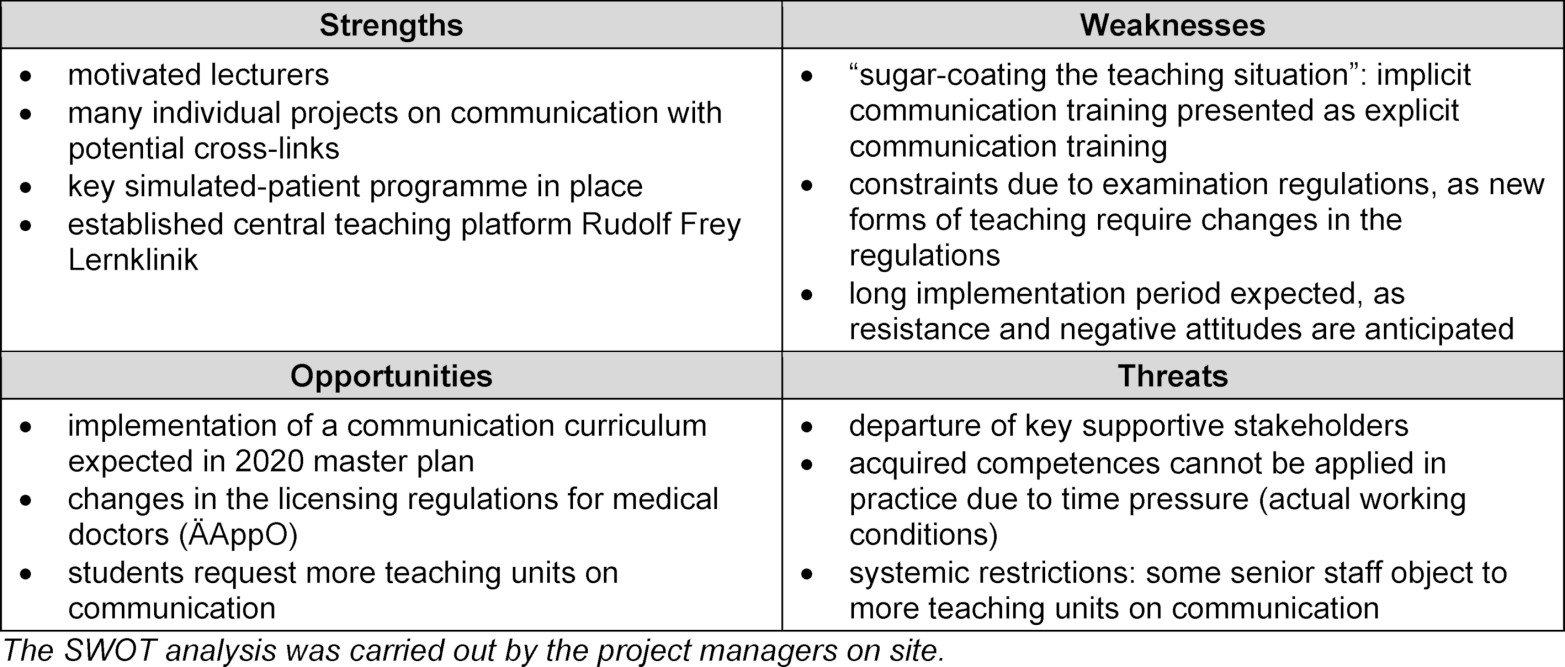
SWOT analysis Mainz location
